# The sequence of a male-specific genome region containing the sex determination switch in *Aedes aegypti*

**DOI:** 10.1186/s13071-018-3090-3

**Published:** 2018-10-20

**Authors:** Joe Turner, Ritesh Krishna, Arjen E. van’t Hof, Elizabeth R. Sutton, Kelly Matzen, Alistair C. Darby

**Affiliations:** 1 Centre for Genomic Research, Institute of Integrative Biology, University of Liverpool, Crown Street, Liverpool, L69 7ZB UK; 2 Oxitec Ltd., 71 Innovation Drive, Milton Park, Abingdon, OX14 4RQ UK; 3 IBM Research UK, STFC Daresbury Laboratory, Warrington, WA4 4AD UK; 4 Liverpool School of Tropical Medicine, Pembroke Place, Liverpool, L3 5QA UK; 5 Department of Zoology, University of Oxford, South Parks Road, Oxford, OX1 3PS UK; 6 Sistemic, West of Scotland Science Park, Glasgow, G20 0SP UK

**Keywords:** M locus, *Nix*, Sex determination, Chromosome evolution, Genomics, BAC, PacBio

## Abstract

**Background:**

*Aedes aegypti* is the principal vector of several important arboviruses. Among the methods of vector control to limit transmission of disease are genetic strategies that involve the release of sterile or genetically modified non-biting males, which has generated interest in manipulating mosquito sex ratios. Sex determination in *Ae. aegypti* is controlled by a non-recombining Y chromosome-like region called the M locus, yet characterisation of this locus has been thwarted by the repetitive nature of the genome. In 2015, an M locus gene named *Nix* was identified that displays the qualities of a sex determination switch.

**Results:**

With the use of a whole-genome bacterial artificial chromosome (BAC) library, we amplified and sequenced a ~200 kb region containing the male-determining gene *Nix*. In this study, we show that *Nix* is comprised of two exons separated by a 99 kb intron primarily composed of repetitive DNA, especially transposable elements.

**Conclusions:**

*Nix*, an unusually large and highly repetitive gene, exhibits features in common with Y chromosome genes in other organisms. We speculate that the lack of recombination at the M locus has allowed the expansion of repeats in a manner characteristic of a sex-limited chromosome, in accordance with proposed models of sex chromosome evolution in insects.

**Electronic supplementary material:**

The online version of this article (10.1186/s13071-018-3090-3) contains supplementary material, which is available to authorized users.

## Background

At least 2.5 billion people live in areas where they are at risk of dengue transmission from mosquitoes, principally *Ae. aegypti*, with an estimated 390 million infections per year [1, 2]. Recently, the emergence of chikungunya and Zika viruses further highlights the public health importance of *Ae. aegypti* [3, 4]. Future mosquito control strategies may incorporate genetic techniques such as the sustained release of sterile or transgenic “self-limiting” mosquitoes [5, 6]. Given that only female mosquitoes bite and spread disease, there has been substantial interest in manipulating mosquito sex determination using these genetic techniques and others, including gene drive [7, 8]. Therefore, elucidating the genetic basis for sex determination could, for instance, facilitate production of male-only cohorts for release, or allow transformation of mosquitoes with sex-specific “self-limiting” gene cassettes.

Sex determination in insects is variable, and generally not well understood outside of model species [9]. Unlike the malaria mosquito *Anopheles gambiae* and *Drosophila* species, *Ae. aegypti* does not have heteromorphic (XY) sex chromosomes [10]. Instead, the male phenotype is determined by a non-recombining M locus on one copy of autosome 1 [11–13]. This locus is poorly characterised because its highly repetitive nature has confounded attempts to study it based on the existing genome assembly [14]. The initial 1376 Mb *Ae. aegypti* reference genome was assembled from Sanger sequencing reads in 2007 [15], which are commonly not long enough to span the repetitive transposable elements that comprise a large proportion of the genome [16], and consequently the assembly was relatively low quality [17]. Furthermore, the fact that both male and female genomic DNA was used for genome sequencing reduced the expected coverage of the M locus to one quarter of the autosome 1 sequences, further obscuring candidate M locus sequences [18].

Recently, a team of researchers was nevertheless able to identify *Nix*, a gene with male-specific, early embryonic expression. Knockout of *Nix* using CRISPR/Cas9 results in morphological feminisation of male mosquitoes along with feminisation of gene expression and female splice forms of the conserved sex-regulating genes *doublesex* (*dsx*) and *fruitless* (*fru*), strongly indicating that *Nix* is the upstream regulator of sexual differentiation [14]. The translated *Nix* protein contains two RNA recognition motifs and is hypothesised to be a splicing factor, acting either directly on *dsx* and *fru* or on currently unknown intermediates [19]. A comparison of sexually dimorphic gene expression in different mosquito tissue types also detected male-specific transcripts of *Nix* [20]*.* An ortholog of *Nix* is present in *Ae. albopictus*, but it is not known if the two are functionally homologous [21].

To date, *Nix* has only been characterised as an mRNA transcript. To fully understand this gene’s role in sex determination and to utilise this knowledge for vector control, it is essential to decipher its genomic context. For this purpose, this study identifies and describes the region of the M locus in which *Nix* is located.

## Results

Four BAC clones positive for *Nix* assembled into a single region of 207 kb with no gaps and a GC content of 40.2% (submitted to the NCBI as accession KY849907). The presence of the *Nix* gene in the assembled BACs was confirmed by BLASTN. The whole gene was present in tiled BACs, though not completely within individual BAC clones. Neither *Nix* nor the complete region could be found in the AaegL3 or Aag2 reference genome assemblies. The newly released AaegL5 male assembly contains *Nix* [22], and the assembled BACs aligned to the corresponding region in AaegL5 with > 99.9% identity, spanning a 2899 bp gap in the AaegL5 genome that is comprised mainly of repeats (Additional file 1: Figures S1, S2). While *Nix* was originally identified in the genome-sequenced Liverpool strain [14], PCR revealed that it is exclusively present in male genomic DNA from other geographically varied *Ae. aegypti* populations (Additional file 1: Figure S3), further strengthening the evidence that it is wholly present in the M locus.

**Fig. 1:**
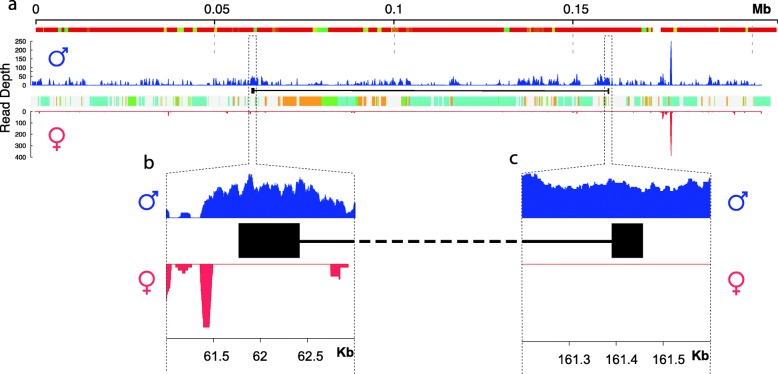
Structure and gene expression of the ~207 kb genomic region containing the *Nix* gene. *Nix* is shown as two black boxes representing the exons, joined by a black line representing the intron. The top track of **a** shows the alignment of the sequence to the corresponding region of the reverse complement of the AaegL5 reference genome assembly, with colours representing percentage similarity (red: 100%; orange: > 90%; green: > 80%). Colours on the central track of **a** represent the classes of repetitive elements (orange: DNA transposons; cyan: Gypsy LTRs; green: Ty1/Copia LTRs). Blue histograms represent the coverage of RNA-Seq reads from male samples on the *y* axis; red histograms represent the coverage from female samples. **b** and **c** show enlargements of the first and second exons of *Nix* in the dotted regions in **a**, respectively

The *Nix* gene was found to be made up of two exons with a single intron of 99 kb (Fig. 1). Although large introns are not uncommon in *Ae. aegypti* (average intron length ~5000 bp) [15], this intron is at the extreme end of intron sizes observed (Additional file 1: Figure S4), especially considering the small size of its protein coding regions (< 1000 bp). The gene structure is confirmed by Illumina RNA-Seq data clearly showing reads spanning the intron between the two exons (Fig. 1). RepeatMasker identified approximately 55% of the sequenced region as repetitive, and the intron region of *Nix* as 72% repetitive (Additional file 2: Table S1).

## Discussion

The genomic data from our assembled M locus region show that *Nix* is approximately 100 kb in length - exceptionally long even for an insect, and one of the longest in the mosquito genome. This is particularly unusual because *Nix* is expressed in early embryonic development, before the onset of the syncytial blastoderm stage 3–4 hours after oviposition [14], during which time most active genes have very short introns, or lack them entirely. There is evidence of selection against intron presence in genes expressed in the early *Ae. aegypti* zygote [23]. In *Drosophila*, the majority of early-expressed genes have small introns and encode small proteins, suggesting that selection has favoured high transcript turnover during early embryonic development due to the requirement for short cell cycles and rapid division [24]. It might therefore be expected that selection would limit the *Nix* intron’s expansion to preserve efficient transcription in the zygote.

One possible explanation is the expansion of repetitive DNA. The RepeatMasker results reveal that the *Nix* region contains a high number of repetitive sequences, especially retrotransposons (Fig. 1, Additional file 2: Table S1). The M locus has accumulated repeats in between protein-coding DNA in a manner characteristic of a sex chromosome, which are prone to degeneration by Muller’s ratchet due to the lack of recombination [25–27]. For instance, repetitive sequences comprise almost the entire *Anopheles gambiae* Y chromosome, and these repetitive sequences show rapid evolutionary divergence [28]. Similarly, certain Y chromosome genes of the plant *Silene latifolia* have much larger introns than their X chromosome copies due to the insertion of retrotransposons [29]. A more extreme version of this phenomenon is seen in *Drosophila,* where some Y chromosome genes, such as those involved in spermatogenesis, have gigantic repetitive introns, sometimes in the megabase range, that consequently make them many times larger than typical autosomal genes [30, 31].

It is therefore possible that the lack of recombination may pose constraints on the structure of the M locus, and in the absence of strong selection the *Nix* gene has degenerated outside the coding regions. Non-recombining sex loci such as the *Ae. aegypti* M locus may represent an evolutionary precursor to differentiated sex chromosomes, which are thought to emerge when sexually antagonistic alleles accumulate on either chromosome and favour reduced recombination between the two homologs, eventually leading to degeneration and loss of genes on the proto-Y [32]. Recent data appears to show that recombination is reduced along chromosome 1 even outside of the M locus [33], while the fully differentiated *Anopheles* X and Y chromosomes still display some degree of recombination with each other [28]. Thus, *Ae. aegypti* may be “further along” this evolutionary trajectory than previously assumed. The presence of additional repeats in our BAC assembly, which was obtained from the My1 mosquito strain, compared to the corresponding region in the AaegL5 genome assembly obtained from the Liverpool strain, suggests that the M locus may vary between strains outside of the *Nix* exons. Future work could investigate the population-level variation in the size and content of the M locus.

The *Ae. aegypti* M locus provides an intriguing example of the complexity of evolutionary forces acting on sex chromosomes, and further study of the locus will contribute to understanding the evolution of sex determination in insects and address general questions about the factors impacting gene and genome length. Importantly, these may also yield insights that can be applied to increase the efficiency of genetic strategies for vector control.

## Methods

### BAC library construction

A BAC library was constructed using living DH10b phage resistant *Escherichia coli* transfected with the pCC1BAC low copy number vector and *Ae. aegypti* genomic DNA from a DNA pool of approximately 50 sibling males (Amplicon Express, USA). Average insert size was 130 kb and the estimated coverage was ~5× for autosomal regions (~2.5× for sex specific regions). The male siblings were from one family from the My1 laboratory strain originating in Jinjang, Kuala Lumpur, Malaysia in the 1960s (described in [34]), after five generations of full-sib mating. Superpools and matrixpools were supplied to allow PCR based screening of the BAC library.

### BAC library screening, isolation and sequencing

The BAC library was PCR screened using primers (Nix1F 3'-TTG AGT CTG AAA AGT CTA TGC AA-5', Nix1R 3'-TCG CTC TTC CGT GGC ATT TGA-5', Nix2F 3'-ACG TAG TCG GCA ACT CGA AG-5', Nix2R 3'-CTG GGA CAA ATC GAA CGG AA-5') based on the complete coding sequence of *Nix* (GenBank: KF732822). The first primer set was also used to screen for *Nix* in the genomic DNA of six male and six female individuals each from two wildtype *Ae. aegypti* strains.

Screening of the library resulted in four positive clones - two for each primer pair. These BAC clones were propagated, extracted using a Maxiprep kit (Qiagen, Hilden, Germany), pooled before SMRTbell library preparation (PacBio, Menlo Park, CA, USA), and sequenced on a single SMRTcell using P6-C3 chemistry on the PacBio RS II platform (PacBio, USA).

### Data analysis

The sequence data was trimmed to remove vector sequences and adaptors prior to assembly with the CANU v1 assembler [35], followed by sequence polishing with QUIVER.

BLASTN was used to assess the uniqueness of the assembled *Nix* region compared to the *Aedes aegypti* Liverpool reference genome AaegL3 and the newer Aag2 cell line assembly. Illumina data generated from male and female genomic DNA (accession numbers SRX290472 and SRX290470) and RNA (accession numbers SRX709698-SRX709703) were mapped to a combined reference containing the assembled *Nix* region added to the AaegL3 genome. DNA samples were mapped with BOWTIE 2.2.1 (using default parameters with -I 200 and -X 500) and RNA-Seq data with TOPHAT 2.1.1 version (using default parameters). RNA-Seq data was processed using the CUFFLINKS 2.2.1 pipeline to look for potential genes and male/female specific expression from the region.

Genes were predicted using AUGUSTUS and the *Aedes aegypti* model [15], repetitive regions described using REPEATMASKER 4.0.6 and the *Ae. aegypti* repeat database.

## Additional files

Additional file 1: **Figure S1.** Alignment of the 207 kb BAC region to the corresponding region in the AaegL5 male reference assembly. **Figure S2.** Alignment of the 207 kb BAC region to chromosome 1 of the AaegL5 male reference assembly. **Figure S3.** PCR screening of the M locus gene *Nix* in male and female DNA of wild type *Aedes aegypti* strains. **Figure S4.** Intron size distribution in *Aedes aegypti* Liverpool reference genome AaegL3. (PDF 249 kb)

Additional file 2: **Table S1.** Types and abundance of repeats in the 207kb assembled M locus region and 99 kb *Nix* intron, identified by RepeatMasker using the *Aedes aegypti* repeat library. (XLSX 10 kb)

## Abbreviations

AaegL#: *Aedes aegypti* Liverpool (LVP) strain reference genome assembly, version #
BAC: Bacterial artificial chromosome
CRISPR/Cas9: Clustered Regularly Interspaced Short Palindromic Repeats/CRISPR-associated protein-9 nuclease
LTR: Long terminal repeat
PCR: Polymerase chain reaction
RNA-Seq: RNA sequencing
WHO: World Health Organisation
