# Supplementary material for: The sequence of a male-specific genome region containing the sex determination switch in Aedes aegypti
Source: Parasit Vectors. 2018 Oct 20;11:549. doi: 10.1186/s13071-018-3090-3 (PMC6195999; doi:10.1186/s13071-018-3090-3)
Supplement: Supplementary file 1 — Figure S1. Alignment of the 207 kb BAC region to the corresponding region in the AaegL5 male reference assembly. Figure S2. Alignment of the 207 kb BAC region to chromosome 1 of the AaegL5 male reference assembly. Figure S3. PCR screening of the M locus gene Nix in male and female DNA of wild type Aedes aegypti strains. Figure S4. Intron size distribution in Aedes aegypti Liverpool reference genome AaegL3. (PDF 249 kb) [file 13071_2018_3090_MOESM1_ESM.pdf]

Additional file 1

Joe Turner, Ritesh Krishna, Arjen E. van ‘t Hof, Elizabeth R. Sutton, Kelly Matzen, Alistair C. Darby.

The sequence of a male-specific genome region containing the sex determination switch in *Aedes aegypti.*

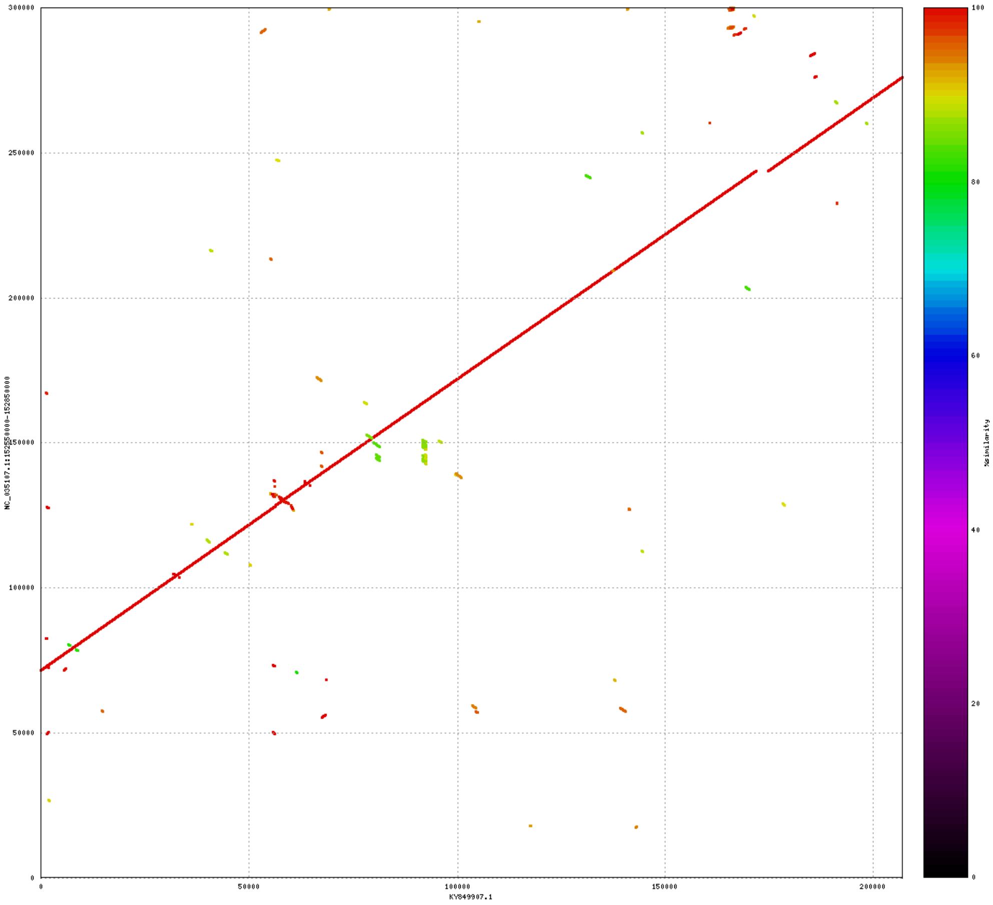

Figure S1: Alignment of the 207 kb BAC region to the corresponding region in the AaegL5 male reference assembly. The query BAC sequence is on the *x* axis (KY849907.1) and the reverse complement of the reference genome sequence is on the *y* axis (NC_035107.1:152550000-152850000, indicating coordinates 152,550,000-152,850,000 on chromosome 1). Plot lines are coloured by percentage similarity. Alignment and plot generated using MUMMER 4.0.0 [1].

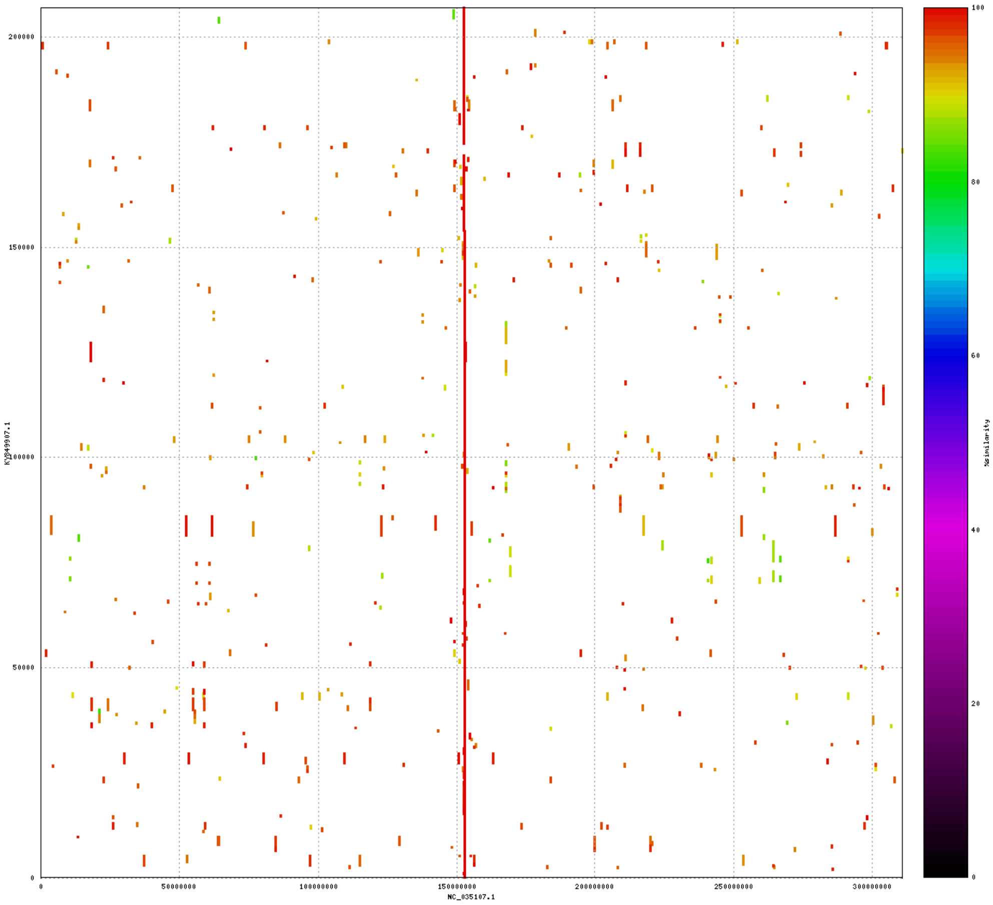

Figure S2: Alignment of the 207 kb BAC region to chromosome 1 of the AaegL5 male reference assembly. The query BAC sequence is on the *y* axis (KY849907.1) and the reverse complement of the reference genome sequence is on the *x* axis (NC_035107.1). Plot lines are coloured by percentage similarity. Alignment and plot generated using MUMMER 4.0.0 [1].

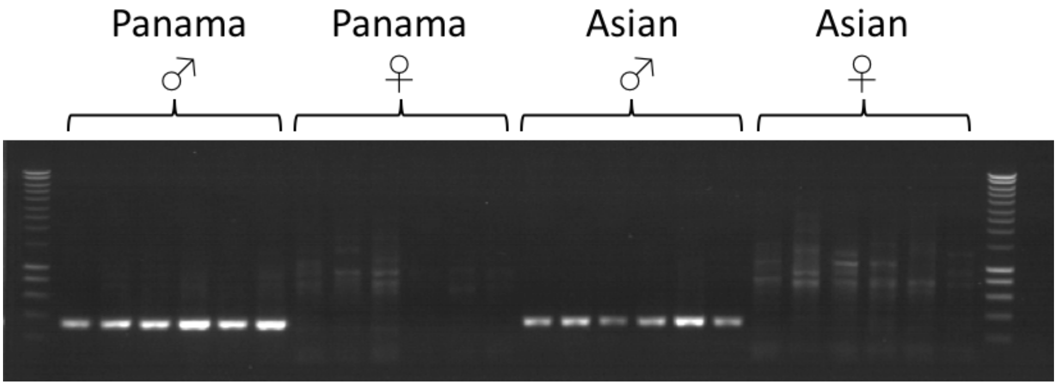

Figure S3: PCR screening of the M locus gene *Nix* in male and female DNA of wild type *Aedes aegypti* strains. Primers used were Nix1F (3’-TTGAGTCTGAAAAGTCTATGCAA-5’) and Nix1R (3’-TCGCTCTTCCGTGGCATTTGA-5’), targeting *Nix* exon 1.

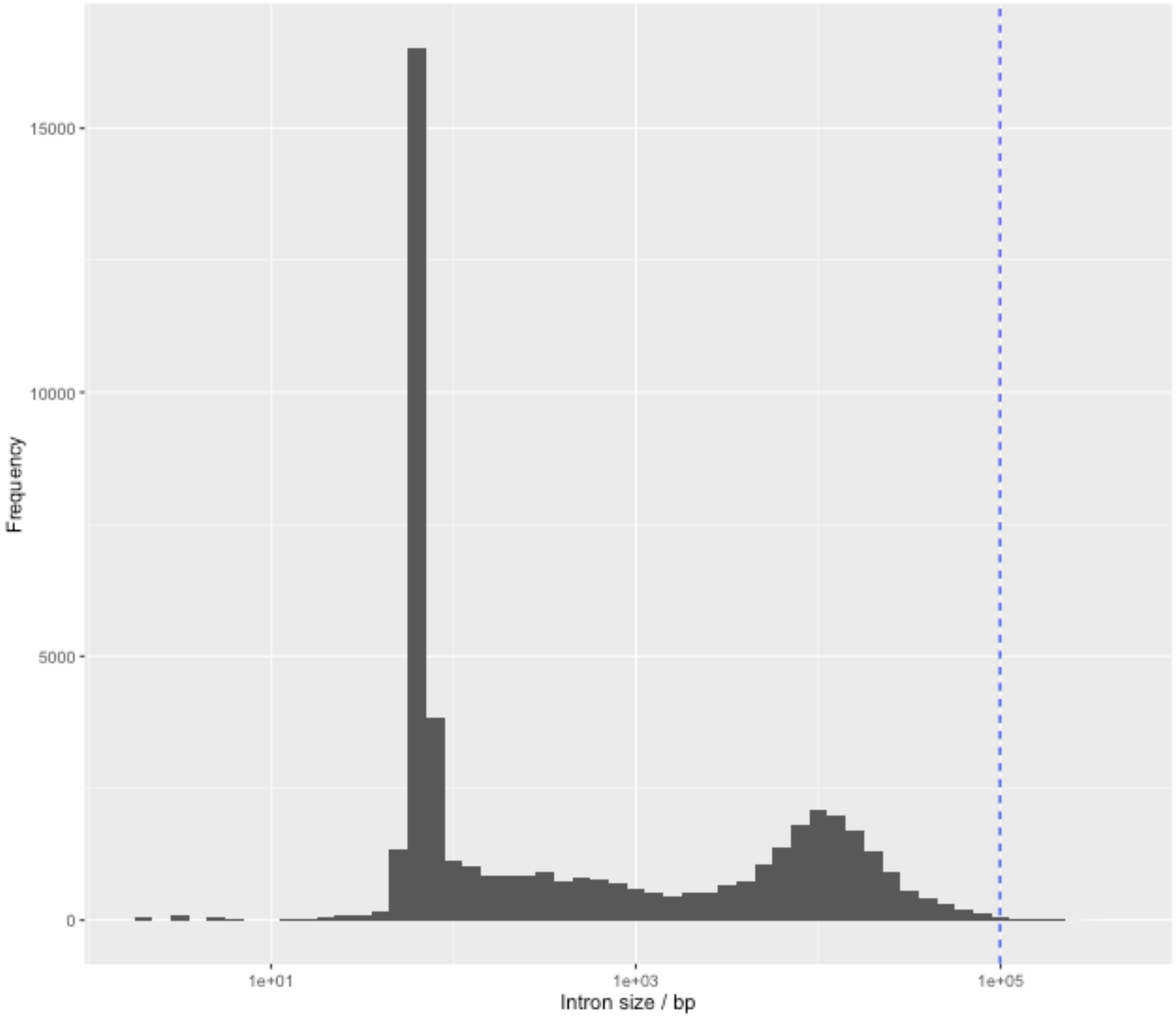

Figure S4: Intron size distribution in *Aedes aegypti* Liverpool reference genome AaegL3. Blue dashed line indicates the size of the *Nix* intron relative other introns. X axis is transformed by log_{10}.

References

1. Kurtz S, Phillippy A, Delcher AL, Smoot M, Shumway M, Antonescu C, et al. Versatile and open software for comparing large genomes. Genome Biol. 2004;5:R12.
